# Untargeted Metabolomics Reveals Metabolic Reprogramming Linked to HCC Risk in Late Diagnosed Tyrosinemia Type 1

**DOI:** 10.3390/metabo16010021

**Published:** 2025-12-24

**Authors:** Anna Sidorina, Cristiano Rizzo, María Jesús Leal-Witt, Carolina Arias, Ignacio Cortés, Verónica Cornejo, Elisa Sacchetti, Giulio Catesini, Sara Boenzi, Carlo Dionisi-Vici, Karen Fuenzalida

**Affiliations:** 1 Division of Metabolic Diseases and Hepatology, Bambino Gesù Children’s Hospital, IRCCS, 00165 Rome, Italy; cristiano.rizzo@opbg.net (C.R.); elisa.sacchetti@opbg.net (E.S.); giulio.catesini@opbg.net (G.C.); sara.boenzi@opbg.net (S.B.); carlo.dionisivici@opbg.net (C.D.-V.); 2 Laboratory of Genetic and Metabolic Diseases, Institute of Nutrition and Food Technology INTA, University of Chile, Av. El Libano 5524, Santiago 7830490, Chile; mj.leal@inta.uchile.cl (M.J.L.-W.); carias@inta.uchile.cl (C.A.); icortes8@santotomas.cl (I.C.); vcornejo@inta.uchile.cl (V.C.)

**Keywords:** hereditary tyrosinemia type 1, hepatocellular carcinoma, untargeted metabolomics, Wnt/β-catenin signaling

## Abstract

**Background/Objectives**: Tyrosinemia type 1 (HT-1) is a treatable inherited disorder characterized by disrupted tyrosine metabolism, leading to severe liver, renal, and occasionally neurological dysfunction. Early diagnosis by newborn screening markedly reduces the risk of hepatocellular carcinoma (HCC), the most serious complication. A deeper understanding of HT-1 pathophysiology is necessary to prevent disease complications and improve diagnostic and therapeutic strategies. This study explored the untargeted serum metabolomic profiles of HT-1 patients. **Methods**: High-resolution untargeted metabolomics coupled with liquid chromatography was applied for serum analysis of 16 late-diagnosed Chilean HT-1 patients on nitisinone (NTBC) therapy and 16 age- and sex-matched controls. The statistically significant up- and down-regulated features were used for annotation and association with different metabolic pathways. **Results**: Untargeted metabolomics revealed 1066 features significantly changed in HT-1 patients. Increased metabolites included aromatic compounds, medium- and long-chain acyl-carnitines, bile acids (prevalently taurine-conjugated), indole-based compounds, modified nucleosides and nucleobases. Decreased metabolites were mainly related to lipid class, including lysophosphatidylcholines, lysophosphatidic acids, long-chain fatty acids, and acylglycerols. **Conclusions**: Untargeted metabolomics showed perturbation of tyrosine- and tryptophan-related pathways and described a novel HT-1 metabolomic pattern demonstrating net dysregulation of lipid and bile acid metabolism in NTBC-treated patients with delay diagnoses. Increased acylcarnitines, taurine-conjugated bile acids, modified nucleobases, and reduced lysophosphatidylcholines overlap with the metabolomic pattern previously reported in Wnt/β-catenin-associated HCC. Although direct mechanistic link cannot be established in this study, these alterations may reflect persistent disease-related metabolic adaptations and warrant further investigation to clarify their potential relevance with long-term complications.

## 1. Introduction

Tyrosinemia type 1 (HT-1) is a rare inherited metabolic disorder caused by a deficiency of the enzyme fumarylacetoacetate hydrolase (FAH), the final enzyme in tyrosine catabolism. FAH deficiency leads to the accumulation of toxic metabolites, including fumarylacetoacetate and succinylacetone (SA), resulting in severe liver and renal dysfunction, and an elevated risk of developing hepatocellular carcinoma (HCC) [1,2,3]. Untreated HT-1 rapidly progresses to liver failure and HCC, posing a significant threat to patient survival [4].

The introduction of nitisinone (2-(2-nitro-4-trifluoromethylbenzoyl)-cyclohexane-1,3-dione, NTBC), a potent inhibitor of 4-hydroxyphenylpyruvate dioxygenase, has revolutionized the management of HT-1 by preventing the formation of these toxic metabolites. When initiated early and combined with dietary management (low-tyrosine and phenylalanine diet supplemented with protein substitutes), NTBC significantly improves survival and reduces the risk of HCC, emphasizing the crucial role of newborn screening [1,5,6]. The best outcomes occurred when NTBC was introduced during the first weeks of life, while patients diagnosed later have an elevated risk of developing HCC [5,6,7,8,9]. Although NTBC has dramatically improved the prognosis of HT-1, long-term complications can still remain. Transcriptome analysis of Fah-deficient mice revealed elevated expression of HCC-related genes in the liver despite NTBC treatment [10]. Furthermore, some patients receiving NTBC may present progressive cognitive impairment, which may be a direct consequence of an increase in tyrosine levels induced by the therapy [11,12,13]. This highlights the need for a better understanding of the long-term metabolic consequences of the disease and its treatment. While the primary biochemical hallmarks of HT-1 and the direct effects of NTBC are well established, characterizing the broader metabolic alterations in treated patients, particularly those with a history of late diagnosis, remains crucial.

Untargeted metabolomics, a powerful approach for the global profiling of small molecules in biological samples, offers an invaluable tool for revealing intricate metabolic dysregulations that extend beyond the immediate consequences of FAH deficiency and NTBC action. Today, most metabolomics studies in HT-1 have focused primarily on disease screening within the broader context of inborn errors of metabolism and therefore restricted their analyses to a narrow set of canonical HT-1 biomarkers, including tyrosine, succinylacetone, 3-(4-hydroxyphenyl)lactate, 4-hydroxyphenylacetate, homovanillate, phenyllactate, and 4-hydroxyphenylpyruvic acid, while largely overlooking broader metabolic alterations [14,15,16,17]. Only limited experimental work in the HT-1 murine model has explored further metabolic changes related to NTBC treatment and evaluated the effects of discontinuing short-term therapy. The NTBC suspension caused perturbations in serum metabolites associated with the glutathione and tryptophan pathways as well as the oxidative stress response [18]. Consequently, a comprehensive characterization and analysis of the HT-1 metabolomic signature is still needed to provide novel insights into disease pathophysiology, identify potential biomarkers for long-term complications such as HCC, and inform personalized therapeutic strategies.

Therefore, the aim of this study was to perform n untargeted, high-resolution mass spectrometry-based characterization of the serum metabolome in NTBC-treated HT-1 patients diagnosed after symptom onset—a real-world, guideline-managed cohort with heterogenous ages and treatment durations—and to compare their metabolic profiles with those of healthy controls, to identify metabolic signatures that may provide insight into long-term disease consequences and potential biomarkers of complications.

## 2. Materials and Methods

### 2.1. Patients

All procedures were conducted in accordance with the ethical standards of the Ministry of Health of Chile and were approved by the Ethics and Scientific Committee review board at the Institute of Nutrition and Food Technology, University of Chile (N°P19/2024). HT-1 patients diagnosed by biochemical and/or genetic testing and in active follow-up (more than 4 outpatient visits per year) were eligible for inclusion in this study. All patients were diagnosed after developing symptoms, with a median age at diagnosis of 9.8 months (range, 2.6–63.4 months). Once the diagnosis was confirmed, they received pharmacological and nutritional treatment consisting of oral administration of NTBC (1 mg/kg/day) in two daily doses, along with a restricted tyrosine and phenylalanine diet supplemented with protein substitutes. All patients included in the study adhered to the treatment regimen. Serum samples were collected during routine follow-up (January 2024) and stored at −80 °C prior to analysis. Sixteen age- and sex-matched healthy subjects were recruited as controls. All samples from patients and controls were collected under fasting conditions, with blood drawn in the morning to minimize circadian variation. Controls were healthy individuals attending outpatient visits or undergoing preoperative evaluation for non-metabolic conditions. Exclusion criteria included abnormal fasting glucose, lactic acidosis, or clinical suspicion of an inborn error of metabolism. Testing of other biochemical, liver, and metabolic parameters was performed during the same outpatient visit period, as indicated in Supplementary Table S1.

### 2.2. Chemical and Materials

The solvents used for analysis and sample preparation were HPLC-grade methanol (Merck KGaA, Darmstadt, Germany), LC-MS-grade acetonitrile, 99% formic acid (Biosolve Chimie, Dieuze, France), and water (Avantor Inc., Radnor, PA, USA). An internal labeled standard (ILS) solution was prepared as 500 µM Tyrosine-D4, 100 µM Phenylalanine-D5, 100 µM Methionine-D3 (Cambridge Isotope Laboratories, Inc., Tewksbury, MA, USA), 50 µM Lysophosphatidylcholine 26:0-D4 (Avanti Polar Lipids, Inc., Alabaster, AL, USA), 480 µM 2-methyl citric acid-D3 (CDN Isotopes, Pointe-Claire, QC, Canada) in 60% acetonitrile in water.

### 2.3. Metabolite Extraction

Samples were thawed on ice before extraction. 30 µL of ILS solution was added to 200 µL of serum. Samples were extracted with 1 mL of extraction solution composed of acetonitrile, methanol, and water (40:40:20). After 5 s of vortexing, samples were placed on ice for 30 min. Then, samples were centrifuged at 4 °C for 10 min at 13,000 rpm. All the supernatants were transferred into glass vials and evaporated. Dried samples were reconstituted in 300 µL of acetonitrile: water (60:40). Serum aliquots of 50 µL of each sample were pooled together, and 200 µL of pooled serum were extracted as described previously and injected repetitively during the sample batch after every 6 samples as quality control (QC), and for the acquisition of fragmentation spectra in data-dependent mode (dd-MS2).

### 2.4. UHPLC and HRMS

Ultra-high-performance chromatographic separation was performed using an Ultimate 3000 (Thermo Fisher Scientific, Waltham, MA, USA). Chromatographic separation was run on 100 × 2.1 mm, 2.6 µm Accucore-150-Amide column (Thermo Fisher Scientific, Waltham, MA, USA) set to 30 °C with mobile phase A (95% acetonitrile, 0.1% acetic acid, 10 mM ammonium acetate) and B (50% acetonitrile, 0.1% acetic acid, 10 mM ammonium acetate). Chromatographic gradient was as follows: 0–1 min 1% of phase B; 1–10 min 95% phase B; 10–10.5 min 1% phase B until 15 min. The flow rate was 0.5 mL/min, and the injection volume was 2 µL. All samples were injected twice as technical replicates in a random order. QC samples for peak area and RT correction were analyzed repeatedly throughout the entire batch, after every 8 samples. The UHPLC system was coupled with a mass spectrometer Q Exactive (Thermo Fisher Scientific, Waltham, MA, USA), scanning in full-scan MS and dd-MS2 modes. The full-scan MS experiment was set to 70–1050 *m*/*z* range with 140,000 resolution, 3 × 10^6^ AGC target, and maximum IT 100 ms. dd-MS2 mode, used only for pooled samples, was set at 17,500 resolution, 2 × 10^4^ AGC target, maximum IT 35 ms, TopN 12, isolation window 1.5 *m*/*z*, and dynamic exclusion 2.5 s. The features have been fragmented with three different normalized collision energies set to 20, 35, and 50 V. The source ionization parameters were as follows: spray voltage, ±3.5 kV; capillary temperature, 380 °C; sheath gas, 60; auxiliary gas, 20; S-Lens level, 50. The data were acquired independently in both positive and negative modes. Calibration was performed at the start of each batch using calibration mixes (Piercenet, Thermo Fisher Scientific, Waltham, MA, USA) to minimize the ppm error of the intact mass.

### 2.5. Metabolomic Data Processing

The raw data was processed on Compound Discoverer 3.3 (Thermo Fisher Scientific, Waltham, MA, USA) software for deconvolution, compound identification, and statistical analysis. The time effects on the batches were corrected using QC samples, and the internal standards were used to check for the chromatography run stability and outliers. After aligning and grouping chromatographic peaks with the same molecular weight and retention time, the background features were removed from the result table. Metabolite identification was primarily based on accurate mass, retention time, and in silico fragmentation matching, corresponding to level 2 identification confidence. For metabolites not included in our in-house library, confirmation using analytical reference standards was not available; therefore, these annotations should be considered putative. Subsequently, the features were statistically analyzed. Principal component analysis (PCA) and volcano plots were applied to highlight group differences with a set FDR-corrected *p*-value < 0.05, using the Benjamini–Hochberg adjustment method, and a log2 FC > |1| cut-off. Data annotation was performed by searching for matching compounds through online databases (ChemSpider, Metabolika, and mzCloud) and a homemade library (90 compounds). All candidate metabolite structures were verified by the in silico fragmentation and their comparison with the experimental spectrum (FISH score). PCA and box-and-whisker plots were generated using Compound Discoverer 3.3 (Thermo Fisher Scientific, Waltham, MA, USA). Heatmaps and pathway enrichment analysis were performed with the MetaboAnalyst 6.0 web platform [19]. Pearson correlation coefficients (r) and Student test (*t*-test) were calculated using Microsoft Excel 2016 (Microsoft Corporation, Redmond, WA, USA). and JMP^®^ Pro 18.0.2 (JMP Statistical Discovery LLC, Cary, NC, USA).

## 3. Results

### 3.1. Demographics and Clinical Characteristics of Participants

The study design included two groups of participants: 16 patients with tyrosinemia type 1 and 17 healthy individuals (controls). Their demographic and clinical data are summarized in Table 1. The mean age of the participants in the HT-1 group and the healthy control group was 11.3 ± 6.8 and 11 ± 5.6 years, respectively. The duration of nutritional and NTBC treatment in HT-1 patients ranges from 3 months to 21.7 years. Significant differences were found between the patient and control groups in levels of tyrosine, methionine, and phenylalanine. As expected in this pathology, the most notable difference was in tyrosine levels, which showed a 7.7–fold increase compared to the control group. Blood NTBC levels in HT-1 patients were within the therapeutic range for optimal management of HT-1 [20], effectively suppressing the excretion of succinylacetone. In addition, levels of alpha-fetoprotein, a biomarker for hepatocellular carcinoma, were within the normal range (<10 ng/mL) in 4 patients out of 14 (Supplementary Table S1).

### 3.2. Metabolomic Profile of HT-1 Patients

**Figure 1:**
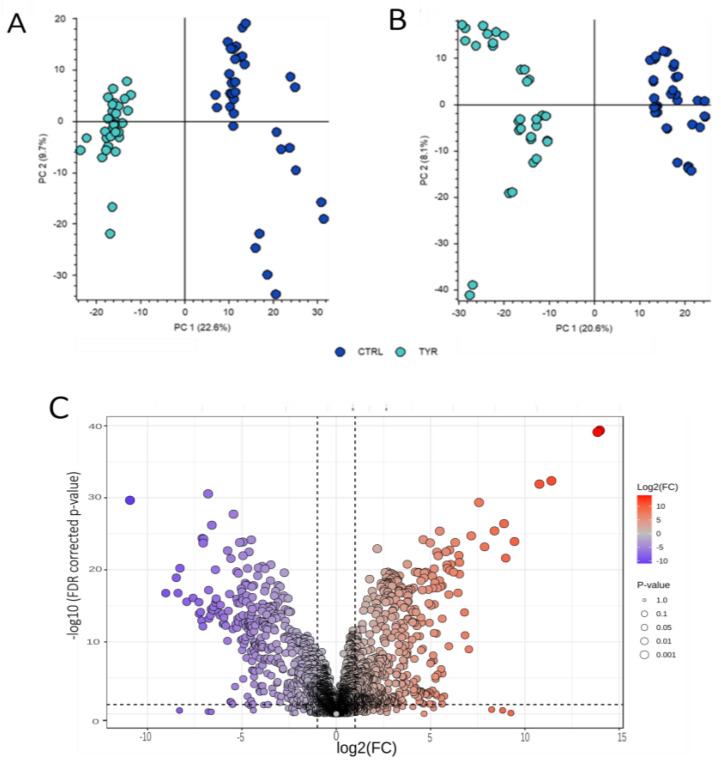
Dimensionality reduction PCA plots of acquired data in positive (**A**) and negative (**B**) mode. Duplicate samples from each control and HT-1 subject are represented in dark blue and light blue dots, respectively. (**C**) Volcano plot of all significantly dysregulated features between control and HT-1 patients with set *p*-value < 0.05 (FDR adjusted) and log2 FC > 1 cut-off (dashed lines).

After removing background compounds, the untargeted metabolomic experiment yielded 1223 features in positive ionization mode and 1673 features in negative ionization mode. A distinctive separation between HT-1 patients and healthy controls was observed when a linear dimensionality reduction technique was applied to the dataset acquired using both acquisition methods (Figure 1A,B).

Using statistical criteria (FDR adjusted *p*-value < 0.05 and absolute log2 fold change (FC) > 1), 1068 features were identified as significantly dysregulated (Figure 1C), with 142 of these being annotated. Specific compounds that exhibited significant changes but had an absolute log2 FC of less than 1 were also included in the results when they belonged to the same category as compounds showing more significant increases or decreases. For instance, methyl adenosine, with a raw FC of 1.7, was added to the modified nucleobases and nucleosides list.

**Figure 2:**
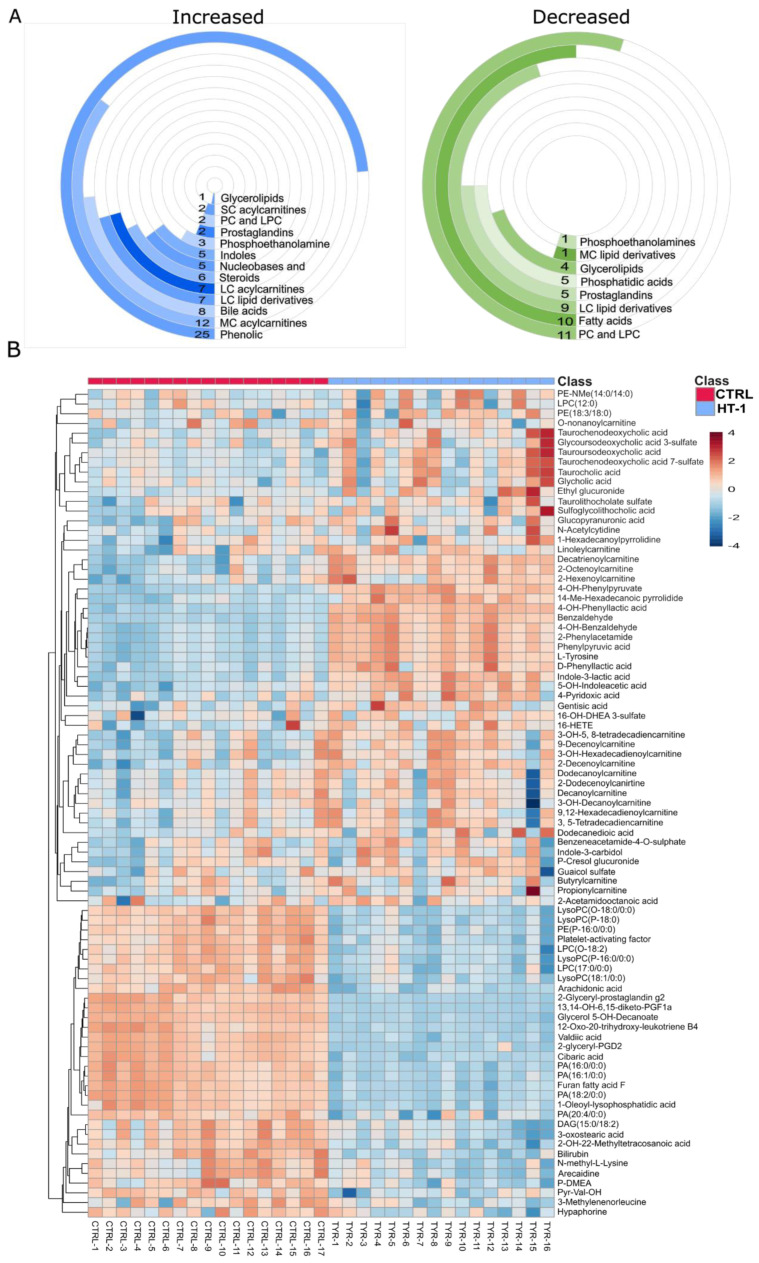
Metabolite dysregulation in HT-1. (**A**) Metabolite Enrichment Analysis of Upregulated and Downregulated Species in HT-1 Patients. The radial bar graph shows the number of dysregulated metabolites classified by chemical classes, either increased or decreased in HT1 patients. SC, Short chain; MC, Medium chain; LC, Long chain; PC and LPC, Phosphatidylcholine and Lysophosphatidylcholine, respectively. (**B**) The heatmap of hierarchical clustering of metabolites significantly altered in HT-1 patients compared to the control group. The analysis includes 94 metabolites identified in HMDB. The color scale represents Z-score normalized abundance.

Enrichment analysis of the differentially dysregulated metabolites in HT-1 patients revealed significant perturbation in aromatic and phenolic compounds, representing a 35.3% increase in metabolites (Figure 2A). General lipids (including steroids, bile acids, fatty acid derivatives, and glycerolipids) accounted for 34.1%; total acylcarnitines comprised 24.7%, and others, like nitrogen-containing compounds, represented 5.8%. The decreased metabolites in HT-1 patients were almost exclusively lipid classes, with lysophospholipids and fatty acids being the predominant groups, together comprising 45% of the reduced lipids. The hierarchical heatmap in Figure 2B displays the normalized abundance (Z-score) of 94 metabolites identified in the Human Metabolome Database (HMDB) that are significantly dysregulated in HT-1 patients compared to healthy control subjects. Among HT-1 patients, no relevant differences in dysregulated metabolites were observed between those with elevated levels of AFP (patient 8, 13, 14 and 16) and those with AFP values within the normal range.

Consistent with this metabolic disease and its therapeutic management, subjects affected by HT-1 exhibited significantly increased tyrosine levels reaching 3.0 log2 FC (8 raw FC), and some downstream tyrosine degradation molecules (Supplementary Table S2). We found that the highest log2 FC were in 4-hydroxyphenyllactic acid (6.0), 4-hydroxyphenylpyruvic acid (4.7), and 3,4-Dihydroxyphenylpyruvic acid (4.6). Other increased tyrosine pathway metabolites were 4-coumaric acid, homovanillin, and 2,5-dihydroxybenzoic acid. Among the indolic compounds, indole-3-lactic acid and 5-hydroxyindoleacetic acid had the highest levels (up to 3.4), while indole-3-carbinol, 5-hydroxyindolepyruvic acid, and 5-hydroxy-1H-indol-6-yl hydrogen sulfate increased up to 1.4 (Supplementary Table S2).

Several increased metabolites were identified in various conjugated or chemically modified forms in HT-1 patients, including nitrogen-containing compounds and glucuronide derivatives. Increased levels of nucleobases and nucleosides were found with methyl or acetyl moiety. Acetylcytidine showed the highest increase (1.1 log2 FC), while methylguanine showed the lowest (1.3 raw FC, 0.4 log2 FC). Glucuronic acid and its derivative, 5-dehydro-4-deoxy-2-O-sulfo-D-glucuronic acid, also increased in HT-1 sera (up to 1.1 log2 FC). Additionally, a compound only identified by its elemental composition (C26H38O9), displaying a fragmentation pattern characteristic of a glucuronide conjugate, showed a remarkable increase (6.9 log2 FC).

The metabolomic profile of the HT-1 group was also characterized by a broad increase in aromatic compounds, which may have originated from gut microbiota activity. Dihydroferulic acid sulfate showed the most pronounced elevation (6.3 log2 FC), followed by benzaldehyde (3.6 log2 FC), trimethyltyrosine (2.4 log2 FC), 4-Pyridoxic acid (2.2 raw FC), cresol derivatives (up to 1.5 log2 FC), and other aromatic and non-annotated compounds (C16H19NO) (Supplementary Table S2).

Decreased non-lipid metabolites included amino acids and dipeptides within the 0.5–1.7 log2 FC range (glutamic acid, methyl lysine, leucylalanine, oxo-prolyl-valine). Additional reductions were observed in N-heterocycle-containing compounds, phosphodimethylethanolamine, and aromatic amines with C16–C18 carbon chains (Supplementary Table S3).

### 3.3. Altered Lipid Homeostasis in NTBC-Treated HT-1 Patients

**Figure 3:**
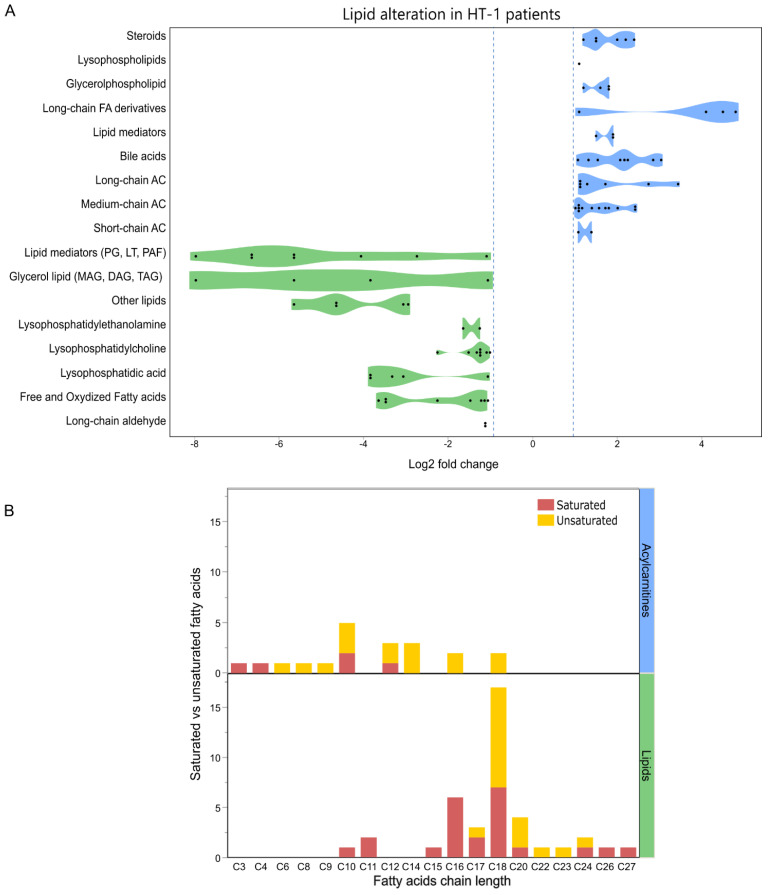
Metabolic Lipid Profiling in HT-1 Patients. (**A**) A violin plot representing the logarithmically normalized fold-change (Log_2_ FC) distributions across diverse lipid classes. (**B**) Comparative analysis of saturated and unsaturated fatty acid composition in acylcarnitines and other lipid species. AC, acylcarnitines.

HT-1 patients exhibited significant alterations in lipid metabolism, characterized by distinct patterns of increased and decreased metabolites (Figure 3). Among elevated species, acylcarnitines were prominently represented, comprising 21 species: 12 medium-chain, 7 long-chain, and 2 short-chain variants. Medium and long-chain acylcarnitines, including saturated and unsaturated forms, showed the most pronounced upregulation, ranging from 1.1 to 3.4 log2 FC in healthy subjects.

Serum bile acids showed increased levels primarily as taurine, taurine-sulfate, and glycine conjugates. Taurine conjugation was predominant, with significant log2 FCs in taurocholic acid (2.8); tauroursodeoxycholic acid (2.2), and taurodeoxycholic acid (3.0). Similarly, taurine-sulfate conjugates, including taurolithocholate sulfate (2.2) and taurochenodeoxycholic acid 7-sulfate (2.1). Glycine conjugation was observed in two bile acids: glycocholic acid (1.5) and glycoursodeoxycholic acid 3-sulfate (1.1). Additional elevated lipid species included various long-chain derivatives: pyrrolidines (C16), cyanolipids (C16, C18), phosphatidylethanolamines (C14, C18), phosphatidylcholine (C18), lysophosphatidylethanolamine (C15), and lipid esters (C14). Significant increases were also observed in lipid mediators such as 12-hydroxy eicosatetraenoic acid (12-HETE), 9-hydroxy-traumatic, dodecanedioic acid, and 2,3-dioctanoylglyceramide as well as steroid-derived compounds (Supplementary Table S3).

In contrast, several lipid categories exhibited significant decreases (Figure 3A). Phospholipids represented the most significantly affected class, particularly lysophosphatidylcholines (8 species, C16–C18, reduced up to 2.3-fold log2) and lysophosphatidic acids (5 species, C16–C20, reduced up to 3.8 log2 FC). Lysophosphatidylethanolamines (C16, C18) showed reductions up to 1.3 log2 FC. Lipid inflammation mediators, including the platelet-activating factor (C16 phosphatidylcholine), decreased by 1.1 log2 FC. Prostaglandins and their metabolites exhibited the most pronounced reductions (4.1–8 log2 FC), along with leukotriene (12-Oxo-20-trihydroxy-leukotriene B4). Free fatty acids (C16–C26) containing hydroxyl and oxo-groups showed reductions within the 1.2–5.6 log2 FC range, including a sulfur-containing C24 fatty acid (docosahexaen-1-ylsulfanyl-acetic acid). Similar compounds showing a decrease included C17/C18 aldehydes, C10 amides, and C18 esters. Glycerol esters with C10, C15, and C18 fatty acids showed marked reductions (Supplementary Table S3).

Fatty acid distribution showed significant variations between increased and decreased lipids. While in decreased lipids, most contain long-chain fatty acids, they are distributed in an almost 1:1 proportion between saturated and unsaturated fatty acids. Elevated acylcarnitines predominantly contain short-chain and medium-chain fatty acids, with unsaturated fatty acids outnumbering saturated fatty acids approximately threefold (Figure 3B).

## 4. Discussion

Tyrosinemia type-1 is a treatable inherited metabolic disease characterized by defective tyrosine catabolism due to fumarylacetoacetate hydrolase deficiency. Although the FDA-approved drug nitisinone (NTBC) and nutritional treatment have dramatically improved patient outcomes, patients with delayed diagnosis (i.e., those not detected by newborn screening) and late treatment initiation remain at elevated risk for hepatocellular carcinoma [5,6]. To comprehensively characterize the metabolic profile of HT-1 patients with late diagnosis, we conducted an untargeted metabolomics analysis of serum samples from HT-1 patients treated with NTBC. Despite the relatively small cohort size, which may limit statistical power and generalizability, this study represents the largest metabolomics analysis reported to date in HT-1, reflecting the rarity of the disease and the challenges associated with enrolling well-controlled patients. This approach aimed to identify dysregulated metabolite clusters and altered biochemical pathways beyond those expected from the underlying pathology and treatment effects, potentially revealing novel insights into disease pathophysiology. Untargeted metabolomic analysis of HT-1 patients with delayed diagnosis and strict adherence to pharmacological and dietary management consistently demonstrated accumulation of metabolites derived from the first steps in the tyrosine degradation pathway, reflected by the NTBC-mediated inhibition of 4-OH-phenylpyruvate dioxygenase. According to this, we found increased levels of tyrosine and 4-hydroxyphenylpyruvic acid in treated patients, as well as their nearby metabolites: 4-hydroxyphenyllactic acid and 4-coumaric acid. Another increased metabolite, identified as 2,5-dihydroxybenzoic acid (gentisic acid), is a catabolic product of homogentisic acid. Its increase in treated patients is not fully understood, as NTBC reduces the generation of the gentisic acid precursor, homogentisic acid [21,22].

Another group of increased HT-1 metabolites, related to NTBC treatment, consisted of indolic compounds derived from perturbation of tryptophan metabolism. As previously demonstrated by Gertsman et al., the characteristic increase in 4-OH-phenylpyruvate following NTBC administration resulted in a shift in tryptophan metabolism toward the indole-pyruvate pathway. As a result, indolic compounds, such as indolepyruvate, indolelactate, and indolecarboxaldehyde, were generated in both host human cells and gut microbiota [23].

The active involvement of gut microbiota in HT-1 was also demonstrated in this study by the presence of numerous aromatic compounds, associated with gut microbiota metabolism, for example: dihydroferulic acid [24,25], benzaldehyde [26], guaiacol sulfate [27], 4-pyridoxic acid [28], cresol and its conjugates [29,30], and others. In recent years, it has become evident that gut microbiota is one of the most critical factors contributing to the pathogenesis of various diseases. Gut microbes transform dietary- and host-derived molecules, generating a diverse group of metabolites with local and systemic effects in psychiatric disorders [31], cancer [28,32], liver diseases [33], and many others [34]. Interestingly, cresol and its conjugates, which increased in HT-1, are microbial metabolites of tyrosine. These metabolites are known as uremic toxins and can enhance the malignant behavior of HepG2 liver cancer cells [35]. The altered gut microbiota metabolites suggest differences in microbial composition or activity between HT-1 patients and control subjects. However, interpreting the underlying causes of these variations is challenging due to the complexity of factors influencing gut microbiota, which include metabolic disturbances associated with the disease, as well as dietary habits, lifestyle, and environmental factors.

HT-1 patients showed an increased level of bile acids. Total bile acid concentration and ratios between individual constituents are widely used as disease biomarkers [36]. Increased circulating bile acids have been associated with obesity [37], type 2 diabetes [38], and various liver diseases, including non-alcoholic fatty liver disease [39], hepatocellular carcinoma (HCC) [40,41], and viral hepatitis [42]. Regarding HT-1, increased bile acids are expected to reflect severe liver dysfunction, a hallmark of HT-1 pathophysiology [43,44]. It was suggested that toxic metabolites (maleylacetoacetate, fumarylacetoacetate, and succinylacetone), accumulating in patients before therapy starts, trigger hepatic complications such as cirrhosis and HCC [43]. Since NTBC was introduced into clinical practice, the risk of severe liver complications has been significantly reduced (although not completely), especially when early diagnosis (newborn screening) and immediate treatment are ensured [5,6]. In our study, all HT-1 patients confirmed their diagnosis late, with a median age of 9.8 months (min–max: 1.5–22 months), therefore facing a higher risk of liver malignancy.

Although most of our patients did not have evidence of HCC at the time of analysis, as indicated by normal levels of AFP in 10/16 and by the absence of suspicious liver imaging changes, their metabolomic profiles were already highly consistent with the metabolomic changes associated with HCC. Along with the general increase in bile acids, which is typical of liver diseases, the entire profile of dysregulated bile acids in HT-1 had a distinct prevalence of taurine-conjugated species, whereas in healthy subjects, glycine conjugation is usually dominant [36,45]. In a recent biomarker-oriented study, Thomas et al. showed that taurine-conjugated bile acids were associated with a higher risk of HCC development [46]. Several metabolomic studies on HCC unrelated to HT-1 have revealed metabolic blood patterns remarkably similar to those observed in our HT-1 cohort. This characteristic pattern included elevated levels of acylcarnitines combined with reduced levels of lysophospatidhylcholines [47,48,49,50,51,52,53,54,55,56,57], elevated levels of modified nucleobases or nucleosides [40,48,57,58] increased hydroxyeicosatetraenoic acid (HETE) [54,59]. The key HT-1 biomarkers, tyrosine and 4-hydroxyphenyllactic acid, were also positively associated with the risk of HCC development [57]. Moreover, metabolic changes might occur up to 10 years prior to HCC diagnosis [57]; therefore, the serum metabolomic profile of HT-1 described in our study suggests that key molecular mechanisms driving HCC development may already be active in NTBC-treated patients with late diagnoses. This observation aligns with a recent report by Neuckermans et al., who performed a comparative whole-transcriptome analysis between HT-1 and Alkaptonuria mouse models [10]. Their study revealed an upregulation of several HCC-associated genes in the HT-1 model compared to the AKP model, which exhibited no hepatic manifestation, supporting the notion that carcinogenic processes may persist in the HT-1 background despite continuous NTBC treatment. The most transcriptionally affected pathways involved liver-specific metabolic processes, lipid homeostasis, and hepatic cholestasis [10]. It should be noted that the present study is restricted to late-diagnosed HT-1 patients due to the unavailability of newborn-screened samples in the HT-1 Chilean cohort. Whether the metabolic pattern observed in the study is already present during the neonatal period (i.e., within the first week of life) remains unknown and warrants investigation, as this could help elucidate the timing of onset of HCC-related metabolomic signatures.

**Figure 4:**
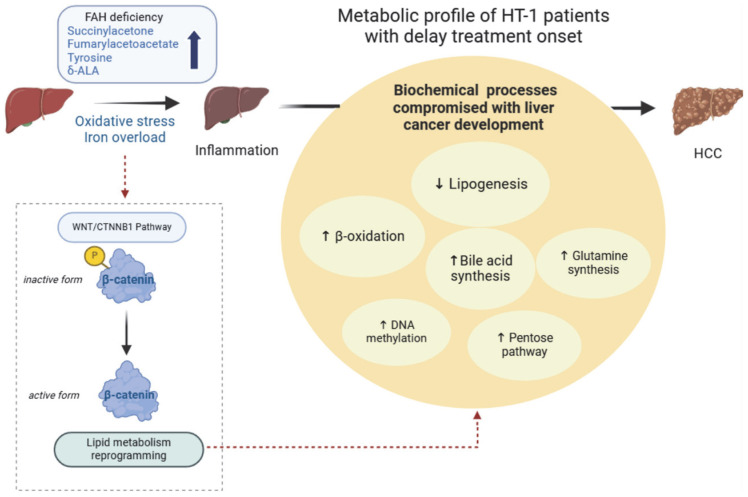
Metabolic profile of HT-1 patients reveals patterns consistent with HCC-associated metabolic reprogramming. Deficiency of FAH leads to the accumulation of several toxic metabolites. Delayed diagnosis and treatment initiation induce chronic liver damage and, consequently, malignant liver cell transformation. Metabolomic analysis of these patients reveals distinctive alterations in lipid homeostasis, similar to those in HCC, driven by activation of the WNT/β-catenin signaling pathway (dashed red lines). These results suggest that despite NTBC therapy, HT-1 patients with late diagnoses may have an ongoing metabolic carcinogenic status.

In addition, the metabolomic profiles observed in HT-1 patients display features that partially overlap with metabolic alterations previously described in Wnt/β-catenin-activated HCC, a well-established driver of HCC pathogenesis [60]. The metabolic reprogramming characteristic of β-catenin-activated HCC leads to significant changes in the lipidome, including a conserved increase in acylcarnitines and ceramides, a decrease in triacylglycerols, and a reduction in phosphatidylcholine production [47,61,62]. Notably, similar alterations were observed in our HT-1 cohort, including generalized increase in acylcarnitines, a reduction in mono-, di-, and triacylglycerols, and a reduction in lysophosphatidylcholines. Reduce circulating free fatty acids in HT-1 serum may also be compatible with enhanced fatty acid oxidation, a characteristic of β-catenin-activated HCC [62]. Beyond lipid metabolism, β-catenin regulation was also connected with DNA and RNA methylation [63,64,65], bile acids metabolism, and liver growth and regeneration [66,67]. While these pathways overlap with several of the metabolic alterations identified in our study, it can be cautiously hypothesized that HT-1 patients diagnosed late, may already present the characteristic biosignatures of metabolic reprogramming seen in β-catenin-driven HCC. Despite NTBC therapy, these metabolic signatures suggest the presence of ongoing liver cellular processes that may be relevant to long-term disease progression, such as HCC development (Figure 4, model proposal). However, whether these alterations reflect dysregulation of Wnt/b-catenin signaling within a broader context of hepatic metabolic adaptation, or are associated with other oncogenic events such as somatic CTNNB1 mutation—reported in approximately 30% of HCC cases [60]—remains unclear and warrants further investigation.

The aberrant activation of the Wnt/β-catenin signaling pathway may be triggered by multiple factors [68,69,70], including the overexpression of several genes. Among them, LPCAT1, which encodes lysophosphatidylcholine acyltransferase 1, has been confirmed to activate the Wnt/β-catenin pathway, contribute to HCC progression, and promote pulmonary metastasis [70]. This enzyme catalyzes the conversion of lysophosphatidylcholine into phosphatidylcholine, and its overexpression could explain the reduced levels of lysophosphatidylcholines associated with HCC [48]. These observations are consistent with our results, showing decreased lysophosphatidylcholines and elevated phosphatidylcholine PC (18:0/8:0) in HT-1 serum. It is worth noting that our experiment was designed for the analysis of small polar analytes. Therefore, phosphatidylcholine species, which carry two fatty acid moieties, may require more specific extraction solvents, generally utilized in lipidomics. The majority of the lipids that changed significantly in our study belong to the polar lipid classes of phospholipids, conjugated bile acids and acylcarnitines, which demonstrated sufficient retention time in the applied HILIC chromatography. The retention of other classes, such as free fatty acids and acylglycerols, on HILIC column was poor, and these lipids may be subject to ion suppression. Therefore, future studies aimed at the comprehensive characterization of the lipidome and proteome in HT-1 patients will be of great interest.

Other phospholipids, reduced in HT-1 patients, were lysophosphatidic acids (LPA). LPA are signaling molecules involved in cell proliferation, migration, and cytoskeletal reorganization. The main pathway of extracellular LPA synthesis consists of the conversion of lysophosphatidylcholines (as well as lysophosphatidylserines and lysophosphatidylethanolamines) into LPA through the enzyme autotaxin (ATX) [71]. The reason for the decreased LPA in HT-1 patients is unclear; it may potentially arise from reduced LPA precursors, such as lysophosphatidylcholines, inhibition of ATX, or other mechanisms. Given the active role of LPA in cell proliferation, LPA signaling has been intensively studied in cancer [72]. However, both cancer broadly and liver cancer specifically typically show intensified LPA-signaling with corresponding increases in ATX and LPA levels—a pattern contradicting our findings in HT-1 patients [73,74,75,76]. Reduced circulating LPA levels have instead been observed in multiple sclerosis and autoimmune encephalomyelitis [77], lecithin cholesterol acyltransferase deficiency [78], psychiatric disorders [79], and are associated with cognitive impairment [80,81].

Some metabolites of the eicosanoid class (one leukotriene derivative and several prostaglandins) were reduced in HT-1. Their common precursor, arachidonic acid, was also deficient in patients, possibly resulting from dietary restrictions. Other reduced metabolites, potentially deriving from dietary restrictions, include glutamic acid, and small peptides commonly observed in HT-1 [82].

## 5. Conclusions

Our metabolomic analysis of NTBC-treated HT-1 patients who were diagnosed late reveals a peculiar metabolic profile that shares similarities with metabolic features previously reported in liver cancer, despite the absence of clinically diagnosed HCC. Rather than implying direct pathway activation, the observed alterations overlap with the metabolic pattern associated with Wnt/β-catenin-related hepatocarcinogenesis and may reflect persistent metabolic adaptations despite NTBC treatment. Overall, our findings provide a metabolic framework for understanding the mechanisms underlying the increased risk of HCC in late-identified HT-1 patients, highlighting the importance of newborn screening to facilitate early diagnosis and treatment and suggesting the validation of novel potential biomarkers for monitoring disease progression and risk stratification of HCC in this vulnerable patient population.

## Figures and Tables

**Table 1:** Demographic and clinical data HT-1 patient and healthy control group.

	Control	HT-1
	(*n* = 17)	*(n* = 16)
Age (year)	11 ± 5.6	11.3 ± 6.8
Sex (Female %)	41	50
Tyrosine (µmol/L)	68 ± 13.6	529 ± 156 *
Phenylalanine (µmol/L)	66 ± 8.7	39.3 ± 15.7 *
Methionine (µmol/L)	27 ± 4.5	20.1 ± 4.8 *
Blood NTBC levels (µmol/L)	-	16.7 ± 5.7
Time of NTBC exposure (years)	-	8.5 (0.25–21.7)
Succinylacetone urine (µmol/mmol creatinine)		N.D.

* Statistical differences were found for tyrosine, methionine, and phenylalanine blood levels (*t*-test *p* < 0.0001). N.D. Not detected.

## Data Availability

The original contributions presented in this study are included in the article/Supplementary Materials. Further inquiries can be directed to the corresponding author(s).

## Supplementary Materials

The following supporting information can be downloaded at: https://www.mdpi.com/article/10.3390/metabo16010021/s1. Table S1: Demographic and follow-up data for HT1 patients, including cross-sectional values of biochemical, metabolic, and liver biomarkers. Mean or median values were calculated for the complete cohort. Treatment time is calculated from the start of nutritional and pharmacological intervention to sample collection. Standard abbreviations: NTBC, Nitisinone in dried blood spot; Met, methionine (plasma); Tyr, tyrosine (plasma); Phe, phenylalanine (plasma); PT, Protrombine Time; ALT, Alanine Transaminase; AST, Aspartate Transaminase; GGT; Gamma Glutamyl Transferase; AlkP, Alkaline Phosphatase, Table S2: Increased metabolites identified in HT-1 serum, Table S3: Decreased metabolites identified in HT-1 serum

## Abbreviations

The following abbreviations are used in this manuscript:

FAH	Fumarylacetoacetate hydrolase
HCC	Hepatocellular carcinoma
HT-1	Hereditary tyrosinemia type 1
NTBC	Nitisinone, 2-(2-nitro-4-trifluoromethylbenzoyl)-cyclohexane-1,3-dione.
SA	Succinylacetone
FC	Fold change
