# Supplementary material for: Untargeted Metabolomics Reveals Metabolic Reprogramming Linked to HCC Risk in Late Diagnosed Tyrosinemia Type 1
Source: Metabolites. 2025 Dec 24;16(1):21. doi: 10.3390/metabo16010021 (PMC12844047; doi:10.3390/metabo16010021)
Supplement: Supplementary file 1 [file metabolites-16-00021-s001.zip › metabolites-4034896-supplementary.pdf]

Table S1. Demographic and follow-up data for HT-1 patients, including cross-sectional values of biochemical, metabolic, and liver biomarkers. Mean or median values were calculated for the complete cohort. Treatment time is calculated from the start of nutritional and pharmacological intervention to sample collection. Standard abbreviations: NTBC, Nitisinone in dried blood spot; Met, methionine (plasma); Tyr, tyrosine (plasma); Phe, phenylalanine (plasma); PT, Protrombine Time; ALT, Alanine Transaminase; AST, Aspartate Transaminase; GGT; Gamma Glutamyl Transferase; AlkP, Alkaline Phosphatase.

Patient #	Sex	Current Age (yrs)	Age at diagnosis (months)	Treatment time (yrs)	NTBC level (µmol/L)	Met (µmol/L)	Tyr (µmol/L)	Phe (µmol/L)	INR	PT (sec)	Bili Total (U/L)	Bili Direct (U/L)	ALT (U/L)	AST (U/L)	GGT (U/L)	AlkP (U/L)	AFP (ng/mL)	Glycemia (mg/dL)
1	M	20.2	11.5	19.2	22.5	19.3	654	39.7	1.1	14.5	0.67	0.35	68	48		112	7.6	88
2	M	4.7	6.8	4.2	10.4	30.9	601	14.2					56	74	39.9		5.2	
3	M	22.0	3.1	21.7	16.5	19.4	625	53.8	1.5	14.0	0.62	0.25	18	26	14	77	3.8	80
4	F	13.2	4.4	12.8	24.0	22.1	590	49.6	1.1	14.8	0.59	0.24	48	47	49	166	8.6	94
5	F	20.4	8.8	19.7	9.8	24.4	669	56.3	1.2	11.3	1.45	0.52	38	36	45	119	3.2	89
6	F	10	63.4	4.7	28.3	18	264	24.0	1.1	12.5	0.82		51	32		381	7.4	82
7	M	9.4	10.7	8.5	16.3	18.7	471	43.8	1.2		0.52	0.21	7	29	75	327	3.8	82
8	F	3.8	27.3	1.6	19.0	14	430	53.0	1.1	14.7	0.55	0.31	30	47	90	375	18.7	89
9	F	7.5	4.7	7.6	15.3	14.7	469	34.5	1.1	15.2	0.34	0.18	26	40	18	470	6.0	92
10	M	17.6	2.6	17.4	21.1	14.9	418	26.4	1.5	14.1	0.52	0.22	31	27	37	97	<2	90
11	F	15.3	6.0	14.7	22.1	23	518	34.3	1.1	13.6	0.74	0.28	18	28	24	165	6.6	87
12	F	18.5	3.3	18.2	16.1	23.8	937	58.1										
13	F	6.4	25.9	4.2	13.5	21.1	584	34.3	1.2	13.4			36	49	60	352	11.6	
14	M	5.5	43.2	1.9	14.4	17.3	452	48.5	1.3	17.2	0.70	0.21	38	60	17	363	39.1	86
15	M	4.5	49.4	0.33	8.3	25.9	354	5.1										
16	M	1.5	15.2	0.25	10.6	14.0	430	53.0	1.1	15.2	0.82	0.46	71	111	156	432	2370	136
Median		9.7	9.8	8.1													7.4	89
Min - Max		1.5 – 22	3.1 – 63.4	0.25 – 21.7													<2 – 2370	
Mean					16.8	20.1	529	39.3	1.2	14.2	0.7	0.3	38.4	46.7	52.1	264.3		91.3
Std Dev					5.7	4.8	157	15.7	0.1	1.5	0.3	0.1	18.8	23	40	143		14.7

Table S2. Increased metabolites identified in HT-1 serum

Name	KEGG PATHWAY or compound class	(HT-1) /(CTRL) Raw FC	Log2 FC	*p*-value (adjusted)
**Metabolites of Tyrosine pathway**				
2,5-Dihydroxybenzoic acid	Tyrosine metabolism, Benzoate degradation, Naphthalene degradation	4,00	2	2.9E-10
4-Coumaric acid	Tyrosine metabolism, Ubiquinone and other terpenoid-quinone biosynthesis, Phenylpropanoid biosynthesis, Biosynthesis of phenylpropanoids, Degradation of aromatic compounds	7	2,8	1.1E-34
Homovanillin	Tyrosine metabolism	6,4	2,7	9.8E-18
4-Hydroxyphenylpyruvic acid	Tyrosine metabolism, Phenylalanine, tyrosine and tryptophan biosynthesis, 2-Oxocarboxylic acid metabolism, Biosynthesis of cofactors	25,4	4,7	2.7E-29
Tyrosine	Tyrosine metabolism, Phenylalanine metabolism, Phenylalanine, tyrosine and tryptophan biosynthesis, Cyanoamino acid metabolism, Aminoacyl-tRNA biosynthesis, 2-Oxocarboxylic acid metabolism, Biosynthesis of cofactors	8	3	4.4E-32
4-Hydroxyphenyllactic acid	Tyrosine metabolism, Ubiquinone and other terpenoid-quinone biosynthesis	62,3	6,0	1.3E-35
3,4-Dihydroxyphenylpyruvic acid	Tyrosine metabolism	25,1	4,6	2.4E-30
**Aromatic compounds putatively deriving from diet and gut microbiota**				
Trimethyltyrosine	Tyrosine derivative	5,3	2,4	2.0E-06
4-Pyridoxic acid	Vitamin B6 metabolite	4,6	2,2	8.4E-15
Cresol sulfate	Cresol	2,7	1,4	5.1E-03
Cresol-3,6-disulfonate hydrate	Cresol	2,9	1,5	5.1E-03
Cresol glucuronide	Cresol	2,3	1,2	2.4E-02
α-Cyano-4-hydroxycinnamic acid	Hydroxycinnamic acid	11,1	3,5	1.8E-19
2-(Hydroxymethoxy)benzenesulfonic acid	Benzenesulfonic acid	2,6	1,4	1.2E-04
Guaiacol sulfate	Catechol	3	1,6	1.2E-04
2-Hydroxy-3-methylphenyl hydrogen sulfate	Catechol	3,1	1,6	3.3E-02
Dihydroferulic acid sulfate	Phenylpropanoic acid	79,6	6,3	4.8E-19
4-Biphenylyl hydrogen sulfate	Biphenylsulfate	5,5	2,5	3.0E-03
C16H19NO	Biphenyl-compound	32,6	5,0	1.0E-17
3-formylsalicylic acid	Naphthalene degradation	4,8	2,3	1.6E-14
4-hydroxyphenylglyoxylate	Monobactam biosynthesis, Aminobenzoate degradation	2,8	1,5	1.5E-12
Benzaldehyde	Toluene degradation, Aminobenzoate degradation	11,8	3,6	1.1E-26
**Indolic compounds**				
Indole-3-carbinol	Indole derivative	2,8	1,5	2.5E-03
5-Hydroxyindolepyruvic acid	Tryptophan metabolism	2,8	1,5	2.6E-05
5-Hydroxyindoleacetic acid	Tryptophan metabolism, Serotonergic synapse	9,9	3,3	3.6E-24
Indole-3-lactic acid	Tryptophan metabolism	10,2	3,4	1.3E-35

5-Hydroxy-1H-indol-6-yl hydrogen sulfate	Indole derivative	2,6	1,4	9.6E-08
**Bile acids**				
Glycocholic acid	Primary bile acid; Primary bile acid biosynthesis, Secondary bile acid biosynthesis, Bile secretion, Cholesterol metabolism	2,9	1,5	1.2E-03
Tauroursodeoxycholic acid	Secondary bile acid	4,7	2,2	9.6E-06
Taurodeoxycholic acid	Secondary bile acid	8,2	3,0	3.9E-06
Sulfoglycolithocholic acid	Secondary bile acid	2,5	1,3	2.6E-02
Taurocholic acid	Primary bile acid; Primary bile acid biosynthesis, Secondary bile acid biosynthesis, Taurine and hypotaurine metabolism, Metabolic pathways, Bile secretion, Cholesterol metabolism	7,2	2,8	2.2E-04
Glycoursodeoxycholic acid 3-sulfate	Secondary bile acid	2,1	1,1	3.4E-05
Taurolithocholate sulfate	Secondary bile acid; Bile secretion	4,5	2,2	2.9E-04
Taurochenodeoxycholic acid 7-sulfate	Primary bile acid; Primary bile acid biosynthesis; Secondary bile acid biosynthesis; Bile secretion; Cholesterol metabolism	4,2	2,1	1.6E-07
**Acylcarnitines**				
Propionylcarnitine (C3)	Short-chain acylcarnitine	2,2	1,1	5.7E-07
2-Octenoylcarnitine (C8:1)	Long-chain acylcarnitine	6,7	2,7	2.9E-15
Butanoylcarnitine (C4)	Short-chain acylcarnitine	2,4	1,3	1.6E-07
9-Decenoylcarnitine (C10:1)	Medium-chain acylcarnitine	3,3	1,7	1.6E-09
Decatrienoylcarnitine (C10:3)	Medium-chain acylcarnitine	10,8	3,4	2.2E-22
Decanoylcarnitine (C10)	Medium-chain acylcarnitine	2,2	1,1	6.9E-03
2-Dodecenoylcarnitine (C12:1)	Medium-chain acylcarnitine	2,2	1,1	3.5E-04
3, 5-Tetradecadiencarnitine (C14:2)	Long-chain acylcarnitine	3	1,6	2.3E-06
Dodecanoylcarnitine (C12)	Medium-chain acylcarnitine	2,1	1,1	5.4E-03
Linoleyl carnitine (C18:2)	Long-chain acylcarnitine	2,1	1,1	1.1E-14
3-hydroxydecanoylcarnitine (C10-OH)	Medium-chain acylcarnitine	2,1	1,1	1.9E-02
Dodecadienoylcarnitine (C12:2)	Medium-chain acylcarnitine	3,3	1,7	4.2E-04
Decadienoylcarnitine (C10:2)	Medium-chain acylcarnitine	5,3	2,4	1.7E-12
Hexadecadienoylcarnitine (C16:2)	Long-chain acylcarnitine	2	1,0	2.2E-04
Nonanoylcarnitine (C9:1)	Medium-chain acylcarnitine	2,2	1,1	1.5E-03
C14:3-carnitine (C14:3)	Long-chain acylcarnitine	5,5	2,5	1.8E-09
Hydroxytetradecadiencarnitine (C14:2-OH)	Long-chain acylcarnitine	3,4	1,8	4.4E-08
C8:2-carnitine (C8:2)	Medium-chain acylcarnitine	4	2,0	2.7E-12
3-Hydroxyhexadecadienoylcarnitine (C16:2-OH)	Long-chain acylcarnitine	2,6	1,4	1.8E-07
C10-oxo-carnitine (C10-oxo)	Medium-chain acylcarnitine	2,1	1,1	1.7E-04
2-Hexenoylcarnitine (C6:1)	Medium-chain acylcarnitine	2,6	1,4	7.5E-11
**Modified Nucleobasis and Nucleosides**				
Acetylcytidine	Nucleoside	2,2	1,1	1.4E-10
Methyladenine	Nucleoside	1,8	0,8	1.2E-14
Acetylcytosine	Nucleobase	2	1,0	1.4E-09
Methylguanine	Nucleobase	1,3	0,4	5.5E-05
Methyladenosine	Nucleoside	1,7	0,8	2.7E-19
**Others**				
Ethylenediamine-N,N'-diacetic acid (EDDA)	Glycine derivative	2,6	1,4	4.3E-10
2-Acetamidooctanoic acid	n-acyl-alpha amino acids	3	1,6	5.0E-05
Glucopyranuronic acid (glucuronic acid)	Glucuronic acid	2,1	1,1	4.3E-17
5-Dehydro-4-deoxy-2-O-sulfo-D-glucuronic acid	Glucuronic acid	2,2	1,1	1.6E-13

Acetylglucose	Hexose	5	2,3	2.1E-05
PE-NMe(14:0/14:0)	Monomethylphosphatidylethanolamine, glycerophospholipid. C14-lipid derivative	3,5	1,8	4.0E-04
2,2-Bis(hydroxymethyl)-1,3-propanediyl ditetradecanoate	C14-lipid derivative	2	1,0	5.0E-05
LysoPE(15:0/0:0)	Lysophosphatidylethanolamine	2,1	1,1	2.3E-02
12-HETE	Arachidonic acid derivative	3,8	1,9	2.7E-08
2,3-Dioctanoylglyceramide	Fatty ester	2,8	1,5	9.0E-08
1-(14-Methylhexadecanoylpyrrolidine	C16-lipid derivative	22	4,5	2.7E-23
Type III cyanolipid 16:0 ester	C16-lipid derivative	28,1	4,8	4.2E-27
1-Hexadecanoylpyr rolidine	C16-lipid derivative	2,2	1,1	3.5E-6
Type IV cyanolipid 18:1(11Z) ester	C18-lipid derivative	16,8	4,1	7.1E-14
16α-Hydroxydehydroepiandrosterone 3-sulfate	Steroid compound	3,9	2,0	1.2E-04
16,17-Dihydroxyandrost-5-en-3-yl hydrogen sulfate	Steroid compound	5,1	2,4	1.6E-05
16,17-Dihydroxyandrost-5-en-3-yl hydrogen sulfate-isom	Steroid compound	4,6	2,2	1.5E-05
Dihydroxy-17-methylandrostan-3-yl hydrogen sulfate	Steroid compound	2,3	1,2	2.4E-05
11-Hydroxyetiocholanolone	Steroid compound	2,8	1,5	1.7E-05
Dihydropregnenolone disulfate	Steroid compound	2,8	1,5	3.6E-04
C26H38O9	Glucuronide compound	123,6	6,9	3.0E-14
PC(18:0/8:0)	Phosphatidylcholine	2,3	1,2	5.8E-10
PE-NMe2(18:2(9Z,12Z)/14:0)	Dimethylphosphatidylethanolamine	3,5	1,8	4.0E-04
PE(18:3(6Z,9Z,12Z)/18:0)	Phosphatidylethanolamine	3,1	1,6	2.8E-04
9-Hydroxy-traumatin	C12-oxo-OH oxydised fatty acid	3,8	1,9	1.3E-07
Dodecanedioic acid	C12-dicarboxylic acid	2,8	1,5	4.0E-04

Table S3. Decreased metabolites identified in HT-1 serum

**Name**	**Compound class**	**(HT-1) /(CTRL) Raw FC**	**Log2 FC**	***p*-value (adjusted)**
**Lipids**				
3-oxostearic acid	(C18-oxo) fatty acid	0,43	-1,2	2.0E-07
8,9-dihydroxy stearic acid	(C18-OH) fatty acid	0,44	-1,2	2.9E-07
Cibaric acid	(C18:3-oxo-OH) fatty acid	0,09	-3,5	9.4E-19
9-hydroxy-16-oxo-hexadecanoic acid	(C16-oxo-OH) fatty acid	0,09	-3,5	3.5E-23
Arachidonic acid	(C20:4) fatty acid	0,46	-1,1	6.9E-14
12-Oxoheneicosa-13,15-dienoic acid	(C21:2-oxo) fatty acid	0,08	-3,6	1.5E-20
2-Hydroxy-22-methyltetracosanoic acid	(C24-OH) fatty acid	0,36	-1,5	1.1E-07
2,3-Dihydroxyhexacosanoic acid	(C26-OH) fatty acid	0,21	-2,3	4.0E-16
Docosahexaen-1-ylsulfanyl-acetic acid	(C24) sulfur containing fatty acid	0,02	-5,6	1.7E-31
Stearoyl lactylate	(C18) fatty acid ester	0,04	-4,6	1.6E-20
9,12,15-octadecatrienal	(C18:3) fatty aldehyde	0,46	-1,1	4.5E-07
9Z-Heptadecenal	(C17) fatty aldehyde	0,46	-1,1	6.9E-07
decanamide	(C10) primary fatty amide	0,13	-2,9	5.4E-28
Glycerol 5-hydroxydecanoate	(C10-OH) monoacylglycerol lipid	0,07	-3,8	3.5E-34

DG(15:0/18:2(9Z,12Z)/0:0)	(C15/C18:2) diacylglycerol lipid	0,48	-1,1	1.3E-05
Diacetoxypropyl pentadecanoate	(C15/C2/C2) triacylglycerol	0,02	-5,6	1.2E-23
TG(18:0(11S-acetoxy)/2:0/2:0)	(C18/C2/C2) triacylglycerol lipid	0,004	-8,0	4.5E-24
1-(1Z-hexadecenyl)-sn-glycero-3-phosphocholine	(C16:1) lysophosphatidylcholine	0,43	-1,2	2.0E-14
LPC O-16:0	(C16) lysophosphatidylcholine	0,4	-1,3	3.6E-19
LPC(P-17:0/0:0)	(C17) lysophosphatidylcholine	0,21	-2,3	6.5E-13
LPC(O-18:2(1Z,9Z))	(C18:2) lysophosphatidylcholine	0,42	-1,3	9.4E-15
LysoPC(P-18:0)	(C18) lysophosphatidylcholine	0,49	-1,0	1.2E-19
LPC(0:0/17:0)	(C17) lysophosphatidylcholine	0,47	-1,1	1.2E-10
LysoPC(O-18:0/0:0)	(C18) lysophosphatidylcholine	0,35	-1,5	2.3E-19
LysoPC(18:1/0:0)	(C18:1) lysophosphatidylcholine	0,42	-1,3	1.4E-12
LPA(18:1/0:0)	(C18:1) lysophosphatidic acid	0,12	-3,1	1.0E-18
LPA(16:1(9Z)/0:0)	(C16:1) lysophosphatidic acid	0,07	-3,8	1.0E-21
LPA(16:0/0:0)	(C16) lysophosphatidic acid	0,07	-3,8	3.8E-24
LPA(18:2(9Z,12Z)/0:0)	(C18:2) lysophosphatidic acid	0,1	-3,3	1.9E-20
LPA(20:4(5Z,8Z,11Z,14Z)/0:0)	(C20:4) lysophosphatidic acid	0,48	-1,1	4.6E-04
LPE(P-16:0/0:0)	(C16) lysophosphatidylethanolamine	0,42	-1,3	5.1E-14
LPE(O-18:1(9Z)/0:0)	(C18:1) lysophosphatidylethanolamine	0,32	-1,6	4.4E-17
Platelet-activating factor	(C16) phosphatidylcholine	0,47	-1,1	4.0E-19
Tetranor-PGE1	(C16) prostaglandin	0,06	-4,1	1.6E-17
Tetranor-PGD1	(C16) prostaglandin	0,01	-6,6	7.1E-32
2,3-Dinor-8-iso prostaglandin F2α	(C18) prostaglandin	0,02	-5,6	4.9E-23
13,14-dihydro-6,15-diketo-PGF1alpha	(C20) prostaglandin	0,004	-8,0	7.8E-29
2-glyceryl-Prostaglandin D2	(C20) prostaglandin	0,01	-6,6	1.7E-26
prostaglandin G2 2-glyceryl ester	(C20) prostaglandin	0,02	-5,6	1.9E-20
12-Oxo-20-trihydroxy-leukotriene B4	(C20) leukotriene	0,15	-2,7	6.1E-15
Undecyl 4-{[1-(dodecyloxy)-3-hydroxy-1-oxo-2-propanyl]amino}-2,3-dihydroxy-4-oxobutanoate	(C11) lipid derivative	0,12	-3,1	1.2E-26
2-Acetoxypropyl (9E)-12-acetoxy-9-octadecenoate	(C18:1) lipid derivative	0,04	-4,6	3.1E-20
**Others**				
Glutamic acid	Amino acid	0,68	-0,6	2.5E-09
Methyl lysine	Amino acid	0,45	-1,2	9.0E-04
Leucylalanine	Amino acid	0,36	-1,5	2.4E-06
Oxo-prolyl-valine	Amino acid	0,31	-1,7	2.6E-05
Piperidone	lactam, N-heterocycle	0,2	-2,3	1.0E-05
Arecaidine	N-heterocycle	0,34	-1,6	1.4E-04
Stachydrine	amino acid and betain derivative, N-heterocycle	0,47	-1,1	6.2E-03
Homostachydrine	indolizine, N-heterocycle	0,19	-2,4	2.9E-11
HMBOA hexose	amino acid and betain derivative, N-heterocycle	0,43	-1,2	7.8E-04
Hypaphorine	amino acid (betain) derivative, N-heterocycle	0,3	-1,7	2.1E-03
Phosphodimethylethanolamine	ethanolamine phosphate	0,2	-2,3	4.8E-06
n-benzyloctadecylamine	Long chain aromatic amine	0,11	-3,2	8.8E-28
N-Butyl-N-hexadecyl-2-methylaniline	Long chain aromatic amine	0,12	-3,1	2.4E-27
